# Synthetic cannabinoids in human post-mortem samples – ADB-BUTINACA and metabolites in three fatalities

**DOI:** 10.3389/ftox.2026.1826767

**Published:** 2026-05-14

**Authors:** Annette Zschiesche, Sophia Köpfler, Cora Wunder, Annekathrin M. Keiler, Johanna Görg, Cleo P. Walz, Barbara Fliss, Tanja Germerott, Volker Auwärter, Laura M. Huppertz

**Affiliations:** 1 Institute of Forensic Medicine, Forensic Toxicology, Medical Center - University of Freiburg, Faculty of Medicine, University of Freiburg, Freiburg, Germany; 2 Hermann Staudinger Graduate School, University of Freiburg, Freiburg, Germany; 3 Furtwangen University of Applied Sciences, Furtwangen, Germany; 4 Institute of Legal Medicine, University Medical Center of the Johannes Gutenberg University, Mainz, Germany; 5 Faculty of Biology, Environmental Monitoring and Endocrinology, TU Dresden University of Technology, Dresden, Germany

**Keywords:** brain tissue, cerebrospinal fluid, fatal intoxications, HepG2 cells, new psychoactive substances (NPS), synthetic cannabinoid receptor agonist (SCRA), toxicological significance score (TSS), vitreous humor

## Abstract

**Background:**

The potent synthetic cannabinoid ADB-BUTINACA (also known as ADB-BINACA) was implicated in three fatal intoxications. Post-mortem samples, including femoral and heart blood, urine, gastric content, bile, vitreous humor, cerebrospinal fluid, and various tissues (brain, kidney, liver, lung, muscle), were analyzed. All cases were mixed intoxications with pregabalin, heroin, ketamine, MDPHP, or ethanol.

**Methods:**

Post-mortem examinations were performed in all three fatalities. Blood and urine were screened by immunoassay, gas chromatography-mass spectrometry (GC-MS), and liquid chromatography-tandem mass spectrometry (LC-MS/MS) for drugs. ADB-BUTINACA was quantified by standard addition in all matrices except vitreous humor and cerebrospinal fluid. For assessing the contribution of ADB-BUTINACA, a Toxicological Significance Score (TSS) was assigned to each case. Metabolite profiles received from analysis of post-mortem matrices were compared with HepG2 cell and human liver microsome data. Additionally, compound stability was assessed over 12 weeks in whole blood.

**Results:**

Femoral blood concentrations of ADB-BUTINACA ranged from 4.2–8.2 ng/mL, and in heart blood from 5.7–11 ng/mL. Brain tissue contained 1.0–6.2 ng/g, and vitreous humor 0.33–2.1 ng/mL. TSS for ADB-BUTINACA of 1 – 3 were assigned for all cases. Heart-to-femoral blood ratios (1.3–1.8) indicated relatively low post-mortem redistribution. Metabolite profile assessment indicated that detection of metabolic biomarkers such as the dihydrodiol could be relevant in urine and bile. Matrix storage at −20 °C is highly recommended to avoid stability issues.

**Conclusion:**

ADB-BUTINACA was detected in all investigated matrices, with the highest concentrations observed in liver, lung, and kidney tissue. The elevated levels in these organs likely reflect their lipophilicity as well as their involvement in absorption (lungs) and metabolism/excretion processes (liver and kidneys). The results of this study may enhance interpretation of toxicological findings in similar cases.

## 1 Introduction

Interpretation of post-mortem toxicological findings remains one of the more complex tasks in forensic toxicology. Concentrations of xenobiotics measured in post-mortem matrices can rarely be directly translated into ante-mortem levels or toxic effects because of numerous post-mortem processes such as redistribution, putrefaction/degradation or matrix-dependent stability (Stephenson et al., 2024; Menéndez-Quintanal et al., 2024; Thieme et al., 2009). These challenges become even more pronounced in cases involving synthetic cannabinoids (SCs), a group of often highly potent and structurally diverse new psychoactive substances (NPS) that continue to emerge on the illicit drug market. Besides designer opioids like nitazenes and orphines, SCs represent one of the most dangerous NPS subclasses with mostly relatively low blood concentrations (Giorgetti et al., 2020). Their rapid evolution, combined with limited pharmacokinetic and clinical data and frequent involvement in polyintoxications further complicates the evaluation of causality in drug-related deaths and systematic risk assessment. Originally developed for pharmacological research, SCs often act as full agonists at the cannabinoid receptors, often with markedly higher *in vitro* potency than Δ9-tetrahydrocannabinol (THC), as shown for ADB-BUTINACA (Cannaert et al., 2020; Sparkes et al., 2022).

**FIGURE 1:**
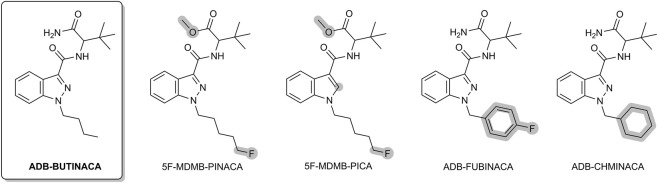
ADB-BUTINACA and several structural analogs, with modifications highlighted in gray, which have been reported in several fatalities.

ADB-BUTINACA (also referred to as ADB-BINACA, ADMB-BINACA or ADMB-BUTINACA) is carrying a tert-butyl carboxamide moiety. It was first reported in Sweden in 2019, has since been detected in seized materials and forensic cases across Europe, and is still very prevalent (European Union Drugs Agency, 2019; Zschiesche et al., 2025b). Case reports, including instances of non-fatal intoxications, suggest a capacity for severe toxic effects (King et al., 2022; WHO, 2022). Its use has been linked to a wide spectrum of adverse effects, including agitation, seizures, hallucinations, tachycardia, cardiovascular collapse, metabolic or respiratory acidosis and reduced consciousness (King et al., 2022). Furthermore, ADB-BUTINACA shows *in vitro* hepatotoxicity (Fan et al., 2024; Zheng et al., 2025). However, comprehensive information on its toxicological profile and post-mortem pharmacokinetics remains limited, with only very few systematically documented fatalities to date (Kang et al., 2025; Kavanagh et al., 2022; Tokarczyk et al., 2023; Zschiesche et al., 2025b). Derivatives such as 5F-MDMB-PINACA (5F-ADB), 5F-MDMB-PICA, ADB-FUBINACA and ADB-CHMINACA (Figure 1) have been implicated in numerous intoxications and fatalities worldwide (Angerer et al., 2017; Giorgetti et al., 2020; Giorgetti et al., 2024b; Groth et al., 2023; Hasegawa et al., 2015; Hermanns-Clausen et al., 2026; Suriaga et al., 2023; Zschiesche et al., 2025a).

Reliable interpretation of SC exposure remains challenging due to scarce data on post-mortem distribution, metabolism, tissue levels and concentrations in alternative matrices, e.g., vitreous humor or cerebrospinal fluid. Many SCs are highly lipophilic substances that undergo extensive metabolism and post-mortem redistribution, which complicates the evaluation of femoral blood results and their toxicological significance (Giorgetti et al., 2020). Comprehensive multi-matrix sampling can therefore be essential, particularly in cases with a higher degree of putrefaction, where conventional matrices are often unavailable (Blavier et al., 2026; Thieme et al., 2009; Truver et al., 2025). *In vivo* and *in vitro* studies have identified key ADB-BUTINACA metabolites (Kavanagh et al., 2022; Kronstrand et al., 2022; Tokarczyk et al., 2023; Zschiesche et al., 2025b), but a systematic comparison with authentic case material is still lacking, but mandatory to better understand post-mortem behavior and toxicological relevance of this compound.

In this study, we present three fatal human intoxications involving ADB-BUTINACA. Concentrations of the parent drug and qualitative assessment of its metabolites were systematically investigated in an extended set of post-mortem samples including femoral and heart blood, urine, gastric content, bile fluid, vitreous humor, cerebrospinal fluid, and multiple tissues (brain, liver, kidney, lung, and muscle). To assess the contribution of ADB-BUTINACA to death, the Toxicological Significance Score (TSS) was employed. The TSS is an established forensic standard for evaluating drug contribution in polydrug fatalities and ranges from 1 (possible contribution to death, alternative cause of death) to 3 (likely or primary cause of death) (Elliott et al., 2018). Complementary *in vitro* experiments with HepG2 cells and pooled human liver microsomes (pHLMs) supported metabolite identification. Additionally, a stability study of ADB-BUTINACA in human whole blood at three different temperatures (−20 °C, 4 °C, 22 °C) was conducted to assess potential degradation over 12 weeks. The study expands the limited toxicological data available for ADB-BUTINACA and provides reference information on its post-mortem distribution and metabolism, contributing to a more robust forensic interpretation.

## 2 Case histories

### 2.1 Case 1

A 38-year-old man visited friends with his partner. He was drunk when they arrived at their friends' house at 4:00 p.m. Both men reportedly consumed “magic tobacco” together. Then, he lay down on the couch. Around 6:00 p.m., rattling respiration sounds were noted. Shortly after, he developed cyanosis and foam at the mouth. Resuscitation efforts were initiated at 6:30 p.m., but remained unsuccessful.

The decedent had a medical history of high alcohol use, consumption of narcotics, epilepsy, diabetes mellitus, and arterial hypertension.

### 2.2 Case 2

A 20-year-old male was found lifeless at home by his mother. She had last contacted him via video chat the previous evening and discovered him the next afternoon lying under a desk. Lay resuscitation was initiated, but emergency services confirmed death on arrival.

Numerous narcotics, new psychoactive substances, drug paraphernalia, and prescribed medications (including risperidone, sertraline, and promethazine) were found in the apartment. The decedent had a history of polysubstance use since adolescence, most recently heroin, and suffered from depression and paranoid delusions.

### 2.3 Case 3

A 53-year-old male was found lifeless on the living-room sofa by his son, who alerted emergency services after noting the body was already cold. Death was confirmed at the scene. The decedent had a history of severe alcohol abuse, multiple comorbidities (esophageal and bowel cancer, chronic obstructive pulmonary disease), and had a stent but did not take prescribed blood thinning medication. According to the son, they had consumed large amounts of alcohol together over 2 days and the deceased had additionally smoked herbal mixtures (“spice”) the evening before death. Numerous empty alcohol containers were present in the apartment.

## 3 Materials and methods

### 3.1 Chemicals

Acetonitrile (ACN, UPLC grade), ammonium formate (10 M) and dimethyl sulfoxide (DMSO) were bought from Sigma-Aldrich (Steinheim, Germany). Deionized water was prepared using a Medica® Pro deionizer from ELGA (Celle, Germany). Formic acid (p.a.) and potassium dihydrogen phosphate were purchased from Carl Roth GmbH (Karlsruhe, Germany). Isopropanol (Prepsolv®) was obtained from Merck (Darmstadt, Germany). Potassium hydroxide pellets were obtained from Honeywell (Seelze, Germany).

The reference standards ADB-BUTINACA and ADB-BUTINACA *N*-3OH butyl were purchased from Chiron AS (Trondheim, Norway), whereas ADB-BUTINACA amide hydrolysis metabolite (MDMB-BUTINACA butanoic acid metabolite), ADB-INACA and the internal standard (IS) d_9_-ADB-BUTINACA were purchased from Cayman Chemical (Ann Arbor, Michigan, USA). ATM4 and ATM4-glucuronide were bought from ASCA GmbH (Berlin, Germany).

Pooled human liver microsomes (pHLMs; 200 donors, 20 mg/mL protein in 250 mM sucrose) were obtained from XenoTech (Kansas City, USA). NADPH-regenerating solutions A (26 mM NADP^+^, 66 mM glucose-6-phosphate, 66 mM MgCl_2_ in H_2_O) and B (40 U/mL glucose-6-phosphate dehydrogenase in 5 mM sodium citrate; reductase activity 0.43 μmol/min × mL), as well as 0.5 M potassium phosphate buffer (pH 7.4), were from Corning (Amsterdam, the Netherlands).

HepG2 cells (ACC 180) were obtained from the German Collection of Microorganisms and Cell Cultures (DSMZ, Braunschweig, Germany). RPMI-1640 medium and phosphate-buffered saline (PBS) were purchased from Sigma-Aldrich (Taufkirchen, Germany). Fetal bovine serum (FBS) and penicillin–streptomycin (100×) were supplied by BioWest (Nuaillé, France).

Roche Diagnostics (Mannheim, Germany) supplied the β-glucuronidase (*Escherichia coli* K12) used for conjugate cleavage.

Phosphate buffer (pH 6) was prepared by dissolving 13.61 g/L potassium dihydrogen phosphate in deionized water. The pH was adjusted by adding a 1 M KOH solution.

Eluent A consisted of 1% acetonitrile, 1% ammonium formate (2 mM), and 0.1% formic acid in deionized water. Eluent B was prepared by mixing acetonitrile with 1% ammonium formate (2 mM) and 0.1% formic acid.

A total of four employees of the Freiburg Forensic Medicine Institute provided venous whole blood voluntarily. The blood was pooled and tested negative for the presence of SCs, their metabolites, or interfering substances before use.

Blank pooled vitreous humor was obtained from the eyes of pigs purchased from a local slaughterhouse and were tested for absence of synthetic cannabinoids or interfering substances before use.

### 3.2 Post-mortem examination and sampling

The autopsy involved a full post-mortem examination, including accurate external examination and internal section, along with the collection of biological fluids and tissues for toxicological analysis. The collected specimens included femoral and cardiac blood, urine, gastric contents (32 g in case 1, 112 g in case 2), bile fluid, vitreous humor, cerebrospinal fluid, as well as brain, kidney, liver, lung and muscle (psoas) tissue.

In case 1, no urine was available for quantification via standard addition, case 2 lacked femoral blood and case 3 gastric content.

The individuals included in this study underwent judicial autopsy to determine the cause of death. Data collection, sampling, and subsequent forensic analyses were requested by the public prosecutor. Publication of data was permitted after the official closure of the cases under the condition to strictly ensure anonymity. All procedures involving human subjects complied with national ethical standards (retrospective study).

### 3.3 General toxicological analysis

General unknown screening was performed using in-house validated techniques: liquid chromatography-high resolution mass spectrometry (LC-HRMS), gas chromatography-mass spectrometry (GC-MS) (both: Supplementary Table S1), immunological screening and targeted liquid chromatography-tandem mass spectrometry (LC-MS/MS).

The femoral blood of case 2 was additionally analyzed using a targeted LC-MS/MS method for designer stimulants including MDPHP and ketamine with a method published elsewhere (Grapp et al., 2020). For case 2, the blood samples as well as the urine sample (with and without enzymatic hydrolysis) were also screened qualitatively for the heroin-markers ATM4 and ATM4-glucuronide (Chen et al., 2014; Maas et al., 2017) after updating a previously published method for the comprehensive detection of opioids (Giorgetti et al., 2024a).

Urinary creatinine was measured using a cobas® 6000 analyzer with c501 module and Creatinine Jaffé Gen.2 reagent (Roche Diagnostics GmbH, Mannheim, Germany).

### 3.4 Standard addition method (SAM)

The standard addition method (SAM) was used to correct for matrix effects common in post-mortem specimens (Hasegawa et al., 2021).

Femoral and heart blood, urine, bile fluid, and homogenized gastric content were diluted with phosphate buffer (pH 6) before SAM application (dilution factors are summarized in Supplementary Table S2) in order to operate in the linear range of the mass spectrometric response.

Tissue samples (brain, kidney, liver, lung, muscle) of approximately 0.5 g were minced with clean surgical scissors, homogenized in a 1.5 mL tube with ceramic beads and 1 mL acetonitrile using a BeadBug homogenizer (Süd-Laborbedarf GmbH, Gauting, Germany), then centrifuged (2,898×g, 10 min). The supernatants were diluted with acetonitrile as detailed in Supplementary Table S2.

Each matrix was processed using 100 µL of the (diluted) sample, with a six-point calibration curve (0, 1, 2, 3, 4, 5 and 6 ng/mL) of ADB-BUTINACA in acetonitrile. Samples were spiked with 1 mL acetonitrile containing d_9_-ADB-BUTINACA (0.5 ng/mL) and 100 µL ammonium formate. Following processing, samples were shaken for 10 min, centrifuged (10 min, 2,898 × g). The supernatant was evaporated to dryness, reconstituted in 100 µL eluent A/B (80:20, *v/v*), and analyzed by LC-MS/MS. SAM was performed in triplicate for each matrix.

### 3.5 External calibration in blank vitreous humor

Vitreous humor and cerebrospinal fluid concentrations were quantified in triplicates using an external calibration curve. For calibration, 200 µL aliquots of porcine vitreous humor (obtained from a local slaughterhouse) were spiked with ADB-BUTINACA at 0.1, 0.25, 0.5, 1, 2.5, and 5 ng/mL. Sample aliquots (200 µL) were prepared and processed identically to the other matrices, using 200 µL of 10 M ammonium formate.

For assessing the limit of detection (LOD) and the limit of quantification (LOQ), 200 µL of pooled porcine blank vitreous humor was spiked with 0.025, 0.05, 0.075, 0.1 and 0.25 ng/mL of ADB-BUTINACA and processed in the same way as the calibrators. Calibration was performed in triplicate. LOD and LOQ were calculated using the software Valistat 2.0 software (Arvecon GmbH, Walldorf, Germany), in accordance with the guidelines of the GTFCh (Society of Toxicological and Forensic Chemistry, Germany) (Peters et al., 2009).

### 3.6 Stability assessment of ADB-BUTINACA in whole blood

The stability of ADB-BUTINACA was assessed in pooled and spiked human whole blood (5 ng/mL, adjusted to reflect concentrations commonly observed in fatalities). Aliquots of 100 µL were stored for up to 3 months light-protected under three conditions: room temperature (22 °C), refrigerated (4 °C), and frozen (−20 °C). Samples were analyzed in duplicate after 0, 1, 3, 7, 14, 21, 28, 42, 56, and 84 days. For each storage condition and time point, a blank sample was included as a negative control. Quantification was performed by external calibration in whole blood, using calibrator concentrations of 0.5, 1, 2, 5, 7, and 10 ng/mL. For LOD and LOQ determination, calibration was performed in untreated whole blood with concentrations of 0.2; 0.3; 0.5; 0.75 and 1.0 ng/mL in duplicates.

For extraction, 5 µL of the deuterated internal standard d_9_-ADB-BUTINACA (250 ng/mL) was added to 100 µL of sample, followed by 1 mL acetonitrile and 100 µL ammonium formate. The mixture was homogenized for 10 min on an overhead shaker and centrifuged (2,898 × g, 10 min). The supernatant was then transferred to an LC vial and evaporated at 40 °C under a nitrogen stream. Reconstitution followed in 100 µL mobile phase A/B (80/20, *v/v*).

### 3.7 Metabolite analysis

#### 3.7.1 In vivo

To detect ADB-BUTINACA metabolites, 100 µL of undiluted post-mortem matrices (supernatants in the case of tissue) were measured once without hydrolysis and once after enzymatic hydrolysis. For this purpose, 1 mL ACN was added to the 100 µL aliquot and was fortified with 100 µL ammonium formate. After shaking and centrifugation (10 min, 2,898 × g), the organic extract was dried under a steam of nitrogen (40 °C) and reconstituted in 100 µL mobile phase A/B (80/20, *v/v*). To hydrolyze the homogenized organ supernatant, 100 µL of the supernatant was evaporated at 40 °C under a nitrogen stream. Then, 100 µL of phosphate buffer (pH 6) and 25 µL β-glucuronidase were added followed by an enzymatic hydrolysis at 45 °C for 1 h. Subsequent processing was carried out in the same manner as for the non-hydrolyzed samples.

#### 3.7.2 In vitro

##### 3.7.2.1 Incubations in pooled human liver microsomes

An *in vitro* pooled human liver microsome (pHLM) assay was carried out to determine the retention times of ADB-BUTINACA phase I metabolites, in accordance with established protocols (Zschiesche et al., 2024). Incubations were performed using ADB-BUTINACA at 10 μg/mL final (1 µL of a 1 mg/mL stock solution in acetonitrile) with pHLM at 1 mg protein/mL final (from 5 µL stock 20 mg/mL), 100 mM potassium phosphate buffer final (pH 7.4, 20 μL, 0.5 M), deionized water (68 µL) and the required enzyme cofactors [NADPH-regenerating solution A (5 µL) and B (1 µL)] at 37 °C for 30 min. Reactions were quenched by adding ice-cold acetonitrile (100 µL) and 10 M ammonium formate (50 µL). The samples were centrifuged for 10 min at 16,550 × g. Afterwards, 5 µL of the organic layer were dried by a gentle steam of nitrogen and reconstituted in 45 µL of eluent A/B (80/20, *v/v*).

##### 3.7.2.2 Incubations in HepG2 cells

HepG2 cells (passage 34) were cultured as monolayers in RPMI-1640 medium supplemented with 10% fetal bovine serum (FBS) and 1% penicillin-streptomycin (P/S) at 37 °C and 5% CO_2_, following a previously published protocol (Zschiesche et al., 2024; 2022). For incubations, cells were grown to ∼60% confluence in 15 mL RPMI-1640 containing 5% dextran-coated charcoal-stripped FBS and 1% P/S. ADB-BUTINACA (200 ng/mL, dissolved in DMSO) was added to the medium, and 1 mL samples were collected at 0 h and 8 days and stored at −20 °C. A solvent control (without drug) was included. For phase I and II metabolite identification, the supernatant was divided into two aliquots. One aliquot underwent enzymatic hydrolysis as described above for *in vivo* samples. Both the hydrolyzed and non-hydrolyzed samples were then processed according to the protocol described for the *in vivo* samples.

### 3.8 High-performance liquid chromatography-electrospray ionization-quadrupole mass spectrometry(HPLC-ESI-QTrap-MS)

The instrument settings and gradient was published elsewhere (Zschiesche et al., 2025b). Briefly, analyses were performed on an Ultimate 3000RS UHPLC system (Dionex, Sunnyvale, USA) coupled to a QTRAP® 6500 triple quadrupole-linear ion trap mass spectrometer (SCIEX, Darmstadt, Germany) in ESI^+^ mode. Separation was achieved on a Kinetex® C18 column (100 × 2.1 mm, 2.6 µm, 100 Å) with an 8.25 min runtime. The injection volume was 10 µL. Table 1 provides the Multiple Reaction Monitoring (MRM) ion transitions of ADB-BUTINACA, the internal standard and the applied ion source parameters.

**TABLE 1:** Optimized mass spectrometric parameters of the MRM ion transitions of ADB-BUTINACA and its deuterated analog.

Analyte	t_R_ [min]	Q1 [Da]	Q2 [Da]	DP [V]	EP [V]	CE [V]	CXP [V]
ADB-BUTINACA	4.41	331.21	201.1	56	10	35	8
			286.2	56	10	21	12
			145.0	56	10	57	16
d_9_-ADB-BUTINACA	4.38	340.27	210.2	80	10	35	15

t_R_: retention time, Q1: *m/z* of the precursor ion, Q3: *m/z* of the fragment ion, DP: declustering potential, EP: entrance potential, CE: collision energy and CXP: cell exit potential.

For metabolite analysis, the MRM-method was expanded to published metabolites (Kavanagh et al., 2022; Kronstrand et al., 2022; Tokarczyk et al., 2023). The MRM-method for the screened metabolites can be found in Supplementary Table S3. The chromatographic peak areas of the metabolites in matrix were normalized to the peak area of the parent substance, ADB-BUTINACA. If the metabolite-to-parent peak area ratio exceeds 1, detection of that metabolite provides greater analytical sensitivity than parent compound detection in the respective matrix.

Data were acquired and processed with Analyst software version 1.6.3 (Sciex, Darmstadt, Germany), and further handled using Microsoft Excel 2007 (Microsoft Corporation, Redmond, WA, USA).

## 4 Results

### 4.1 Autopsy findings

#### 4.1.1 Case 1

The male corpse showed brownish skin discoloration and signs of upper chest venous congestion. Pulmonary and cerebral edema were present. Further findings included cardiac hypertrophy, visceral congestion and hepatomegaly. Furthermore, the autopsy revealed no morphologically definable cause of death. Findings such as cerebral and pulmonary edema, combined with the case history, pointed to a fatal intoxication with drugs or medication.

#### 4.1.2 Case 2

Autopsy revealed marked pronounced cerebral edema, a full urinary bladder, and blood-rich internal organs with shock kidneys. According to the results of the forensic post-mortem examination, the cause of death could not be determined morphologically. Taking into account the previous history and the autopsy findings, intoxication had to be considered as a potential cause of death.

#### 4.1.3 Case 3

Autopsy showed a well-nourished male with upper venous chest congestion, blood-rich organs, and marked cerebral swelling. Endocardial hemorrhages of the left ventricle was noted. The heart was relatively enlarged, with a coronary stent, moderate atherosclerotic changes causing luminal narrowing, and fatty infiltration of the right ventricle. Pulmonary findings included chronic obstructive lung disease and pulmonary hypertension. Additional findings were a moderate generalized adiposity and vascular calcifications. According to the results of the forensic autopsy, there was no morphological cause of death. Considering the medical history, alcohol or drug intoxication had to be considered as a potential cause of death. In the setting of pre-existing cardiac disease and chronic obstructive pulmonary disease, cardiorespiratory failure was considered an alternative cause.

### 4.2 General toxicological analysis

#### 4.2.1 Case 1

Femoral blood contained ADB-BUTINACA, pregabalin (25,000 ng/mL) and ethanol (approx. 0.6‰). Quetiapine was detected in heart blood (260 ng/mL).

#### 4.2.2 Case 2

In femoral blood, alongside ADB-BUTINACA, 50 ng/mL morphine (a main heroin metabolite), 13 ng/mL codeine, 6.8 ng/mL bupropion, 17 ng/mL olanzapine, 12 ng/mL promethazine, approximately 3.3 ng/mL MDPHP, and approximately 4.5 ng/mL ketamine were detected, as well as ketamine metabolites. In heart blood, papaverine (7.5 ng/mL), noscapine (83 ng/mL), codeine (32 ng/mL), dihydromorphine (1.0 ng/mL), morphine (240 ng/mL) and norcodeine (3.7 ng/mL) were detected, but no ATM4, ATM4-glucuronide or 6-acetylmorphine. In hydrolyzed urine, ATM4 (not quantified), codeine (4,100 ng/mL), norcodeine (220 ng/mL), morphine (>20,000 ng/mL) normorphine (3,500 ng/mL), noscapine (99 ng/mL), papaverine (25 ng/mL), dihydromorphine (170 ng/mL), in non-hydrolyzed urine, 6-acetylmorphine (2100 ng/mL), 6-acetylcodeine (66 ng/mL) and ATM4-glucuronide (not quantified) were detected. Creatinine in urine was 90 mg/dL.

#### 4.2.3 Case 3

ADB-BUTINACA was detected along with approximately 2.9‰ alcohol, 0.6 ng/mL Δ9-THC, 5.1 ng/mL Δ9-THC-carboxylic acid and the ester hydrolysis metabolite of MDMB-4en-PINACA in femoral blood. Creatinine in urine was determined at 25 mg/dL.

### 4.3 Quantification of ADB-BUTINACA

The quantitative results for ADB-BUTINACA gained via standard addition in a comprehensive set of post-mortem material are shown in Table 2. The corresponding dilution factors, linear calibration functions and the coefficients of correlation (R^2^ > 0.99 for all matrices) are given in Supplementary Table S2.

**TABLE 2:** Concentrations of ADB-BUTINACA (mean values and standard deviation) in post-mortem matrices determined applying a standard addition protocol in triplicates. The cardiac-to-peripheral blood ratio (C/P) is also provided. The post-mortem interval (PMI) for all cases was 5 days.

Matrix [concentration]	Case 1	Case 2	Case 3
Femoral blood [ng/mL]	4.2 ± 0.25	n.a.	8.2 ± 0.88
Heart blood [ng/mL]	7.8 ± 0.28	5.7 ± 0.11	11.0 ± 0.49
C/P	1.8	-	1.3
Urine [ng/mL]	n.a.	0.26 ± 0.02	0.36 ± 0.04
Gastric content [ng/mL]	3.0 ± 0.40 (total: 96.0 ± 12.0 ng)	1.5 ± 0.06 (total: 160.0 ± 32.0 ng)	n.a.
Bile fluid [ng/mL]	5.0 ± 0.34	12.0 ± 0.41	13.0 ± 1.10
Brain tissue [ng/g]	5.2 ± 0.23	1.0 ± 0.11	6.2 ± 0.13
Kidney tissue [ng/g]	9.2 ± 0.73	3.6 ± 0.12	11.0 ± 0.21
Liver tissue [ng/g]	25.0 ± 1.30	7.1 ± 0.15	54.0 ± 2.10
Lung tissue [ng/g]	5.7 ± 0.18	7.4 ± 0.27	18.0 ± 0.89
Muscle tissue (psoas) [ng/g]	2.0 ± 0.39	1.5 ± 0.03	12.0 ± 0.06

n.a.: not available.

Gastric content: [ng/mL] = concentration in homogenized sample; total (ng) = concentration × sample mass (case 1: 32 g; case 2: 112 g).

The quantitative results of ADB-BUTINACA in vitreous humor and cerebrospinal fluid as determined via external calibration are provided in Table 3.

**TABLE 3:** Concentrations of ADB-BUTINACA (mean values and standard deviation) using an external calibration in blank vitreous humor of pigs in triplicates.

Matrix	Case 1	Case 2	Case 3
Vitreous humor	0.33 ± 0.02 ng/mL	0.79 ± 0.05 ng/mL	2.1 ± 0.14 ng/mL
Cerebrospinal fluid	1.0 ± 0.06 ng/mL	n.a.	4.2 ± 0.19 ng/mL

n.a.: not available.

Table 4 provides the LOD, LOQ and linear range of ADB-BUTINACA in blank porcine vitreous humor.

**TABLE 4:** LOD, LOQ and linear range of the calibration curve used for quantification of ADB-BUTINACA using an external calibration in blank vitreous humor of pigs in triplicates.

Analyte	LOD	LOQ	Linear range	Calibration curve (N = 3)		
				Slope	Intercept	R^2^
ADB-BUTINACA	0.008	0.029	0.1–5	0.3266	0.0215	0.9998

### 4.4 Stability assessment of ADB-BUTINACA in whole blood

**FIGURE 2:**
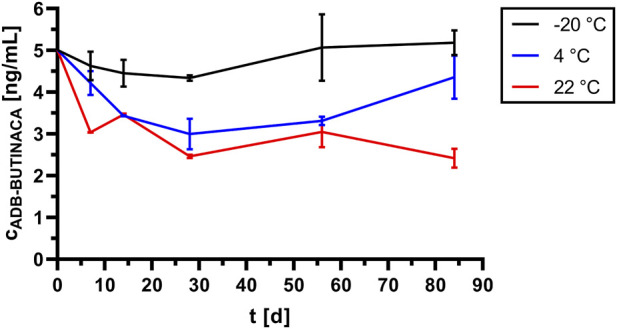
Concentration curve of ADB-BUTINACA (initial: 5 ng/mL) in pooled venous whole blood (mean values from double determination) within 84 days under different storage conditions (−20 °C, 4 °C, 22 °C).

Figure 2 shows the concentration curve of ADB-BUTINACA depending on the storage condition and time in spiked untreated human whole blood.

ADB-BUTINACA showed no significant instability when stored at −20 °C for 3 months. However, when stored in a refrigerator (4 °C), the concentration gradually decreased over the entire period. After 3 months, the concentration decreased by approx. 10% compared to the initial concentration but also showed substantial initial fluctuations (minimum ∼55% around day 14). The concentration decreased significantly within the first few days at 22 °C and continued to decrease throughout the entire period. After 84 days, the concentration had decreased by approx. 50%.

The LOD and LOQ for ADB-BUTINACA in human whole blood were 0.18 and 0.57 ng/mL.

### 4.5 Metabolites

**FIGURE 3:**
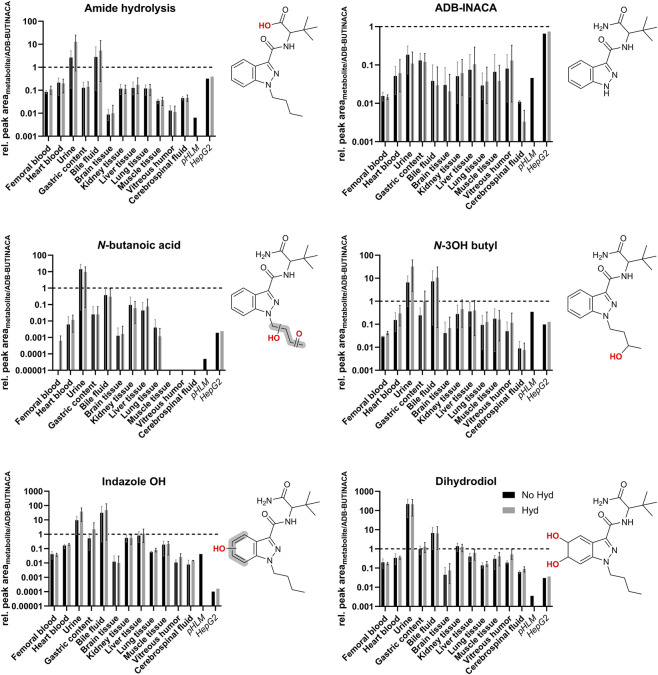
Peak area ratios (logarithmic scale) of six ADB-BUTINACA metabolites in non-hydrolyzed (black, No Hyd) and enzymatically hydrolyzed (gray, Hyd) matrices. Legend applies to all panels. The representation depicts the median values and their range. To facilitate comparison with post-mortem matrices (bold), the outcome of ADB-BUTINACA incubation with pHLM and HepG2 cells is depicted for each metabolite. To improve comparability, the dashed horizontal line indicates a ratio of 1 for each metabolite. For some of the biomarkers, it was not possible to determine the exact site of biotransformation within the structural units highlighted in gray.

The post-mortem samples were analyzed for published phase I and phase II metabolites of ADB-BUTINACA (Gish et al., 2025; Kavanagh et al., 2022; Kronstrand et al., 2022; Zschiesche et al., 2025b). To determine whether the detection of one or more metabolites, in addition to the parent substance, would be advantageous, the ratio of the chromatographic peak areas of the respective metabolite to the peak area of ADB-BUTINACA (relative detectability of metabolites across matrices) was calculated. Figure 3 shows the metabolite ratios of six ADB-BUTINACA phase I metabolites as a function of the post-mortem matrix, with (gray, Hyd) and without enzymatic hydrolysis (black, No Hyd), in semi logarithmic representation. For comparison, the ratios observed after ADB-BUTINACA incubation in the *in vitro* models (pHLM and HepG2 cells) are shown.

**FIGURE 4:**
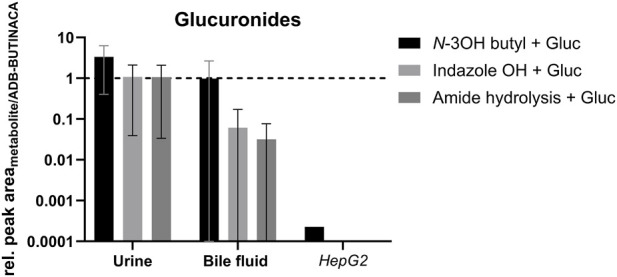
The area ratios (logarithmic scale) are shown for intact, detectable glucuronides of ADB-BUTINACA in non-hydrolyzed urine, bile, and HepG2 incubation supernatants. OH: monohydroxylated species; Gluc: glucuronides.

The dihydrodiol metabolite-to-parent ratio showed relatively high values in urine and bile, averaging 215 and 6, respectively. This ratio was significantly higher than in the other post-mortem matrices examined. The monohydroxylated species (*N*-3-hydroxybutyl and indazole OH) showed comparatively high ratios in urine and bile compared to other matrices. ADB-INACA can be regarded a metabolic side-product resulting from *N*-debutylation. Metabolite ratios were lowest in brain tissue and vitreous humor. The abundance of the *N*-3-hydroxybutyl metabolite increased significantly in urine after hydrolysis, indicating extensive glucuronidation. This effect was less pronounced in the metabolite resulting from amide hydrolysis. Conversely, the dihydrodiol biomarker does not seem to be conjugated to a relevant extent. The metabolite-to-parent ratios of the pHLM and HepG2 incubations were generally below 1 for all six metabolites. Three glucuronides of phase I metabolites were detected directly (as intact molecules). The results for these molecules are shown in Figure 4.

The species monohydroxylated at the *N*-butyl chain appears with the highest abundance in all three matrices. Of all the glucuronides, only this compound was detected in the HepG2 cell incubation. Glucuronides were not detected in liver and kidney tissue.

## 5 Discussion

### 5.1 Concentrations of ADB-BUTINACA – comparison in three cases

Comparatively high concentrations of ADB-BUTINACA ranging from 4.2–8.2 ng/mL (**femoral blood**) and 5.7–11 ng/mL in **heart blood**, suggest recent use. In cases 1 and 3, heart blood levels exceeded femoral blood concentrations. To assess post-mortem redistribution, the central-to-peripheral (C/P) blood ratio was calculated, with values >1 indicating redistribution (Abdelaal et al., 2024). Ratios of 1.8 (case 1) and 1.3 (case 3) are indicative of such processes, while no ratio could be determined in case 2 due to lack of sufficient amounts of femoral blood. The C/P values calculated for case 1 and 3 can be regarded as moderate. For ADB-CHMINACA, C/P values of 1.54–1.75 were reported (PMI = 2 days) (Hasegawa et al., 2015), whereas for MDMB-CHMICA with a PMI of 12 h, a C/P of 1.2 was reported (Gaunitz et al., 2018). In a former case, the C/P ratio of ADB-BUTINACA was 3 with a relatively long PMI of 9 days. In that case, very high ADB-BUTINACA-concentrations (101 ng/mL heart blood, 34.5 ng/mL femoral blood) were reported, likely reflecting a high-dosed intake due to mislabeling of the consumed drug as MDPHP (Zschiesche et al., 2025b). In another report, the death of a police dog after accidental inhalation yielded 8.1 ng/mL in blood (Tokarczyk et al., 2023), comparable to the concentrations observed in the present cases. Kang et al. published two fatal cases with concentrations of ADB-BUTINACA of 14.73 and 13.75 ng/mL in peripheral blood and corresponding cardiac blood concentrations of 30.4 and 11.56 ng/mL, respectively (Kang et al., 2025). In our routine forensic casework (August 2020 till January 2026), a median concentration of ADB-BUTINACA in blood and serum samples of approx. 1.5 ng/mL (mean: ca. 7.6 ng/mL; SD: 24 ng/mL, N = 182) was observed.

In all three cases, high **lung** concentrations (5.7–18 ng/g) were observed, consistent with the reported smoking of “magic tobacco” and “herbal mixtures” in cases 1 and 3. For ADB-CHMINACA, 22.5 ng/g were found (Hasegawa et al., 2015). Inhalative administration is the most common route for SC consumption, bypassing hepatic first-pass metabolism and leading to quick onset of effects. Drug levels in lung tissue may be locally elevated due to direct exposure during smoking, as lipophilic SCs (logP ADB-BUTINACA: 2.76, ChemDraw Professional, ver. 23) readily penetrate lung tissue. Metabolism in the lungs is expected to be marginal compared to systemic clearance.

ADB-BUTINACA was found in moderate concentrations in **gastric content** of all cases, likely reflecting partial swallowing of condensate during smoking (Giorgetti et al., 2020). Post-mortem redistribution from gastric material into the lungs has been suggested after oral uptake (Menéndez-Quintanal et al., 2024), but based on the measured gastric levels (3.0 ng/mL in case 1, 1.5 ng/mL in case 2), such processes did very likely not influence lung tissue concentrations. In addition, agonal aspiration of gastric contents may increase lung drug levels. However, the concentrations measured in gastric content make a major contribution unlikely in the presented cases.

The highest **liver** concentrations of ADB-BUTINACA were observed in cases 1 (25 ng/g) and 3 (54 ng/g), with case 2 also showing a notable level (7.1 ng/g). Owing to their lipophilicity, SCs can accumulate in liver tissue, while extensive hepatic metabolism generally reduces parent drug levels. Enzyme activity of the cytochrome P450 system decreases rapidly after death (≈90% within 48 h), but post-mortem formation of metabolites can still occur over several days due to residual enzyme activity (Menéndez-Quintanal et al., 2024). In the present cases, with a PMI of 5 days, a contribution of post-mortem metabolism in the liver seems likely.

Consistent with reports for other lipophilic SCs, **bile** often contains detectable parent drug concentrations (Schaefer et al., 2020; Walle et al., 2024). ADB-BUTINACA levels in **bile** fluid were among the highest measured: 12 ng/mL (case 2) and 13 ng/mL (case 3). In case 1, however, the bile concentration (5.0 ng/mL) was nearly identical to femoral blood (4.2 ng/mL), an unusual finding that may suggest rapid death with limited bile fluid production (Tokarczyk et al., 2023).

High concentrations of ADB-BUTINACA were also found in **kidney** tissue (3.6–11 ng/g). Like the liver, the kidney’s strong blood supply and the lipophilic nature of SCs promote tissue accumulation. Protein binding may further contribute to tubular reabsorption or persistence in the kidneys (Schaefer et al., 2019).

Low concentrations of ADB-BUTINACA were detected in **urine** (case 2: 0.27 ng/mL; case 3: 0.36 ng/mL; case 1: urine not available). In urine samples of living individuals, parent SCs are rarely detectable due to extensive hepatic metabolism, with mainly polar metabolites persisting. However, parent compounds have repeatedly been reported in post-mortem urine (Kavanagh et al., 2022; Kronstrand et al., 2022; Minakata et al., 2017; Yeter and Erol Öztürk, 2019), likely explained by post-mortem diffusion of lipophilic SCs such as ADB-BUTINACA (Kavanagh et al., 2022; Zschiesche et al., 2025b).

Concentrations of ADB-BUTINACA in the **brain** ranged from 1.0–6.2 ng/g. Due to the brain’s high blood flow, lipid content, and the presumed ability of SCs to efficiently cross the blood-brain barrier, accumulation of the active substance in the brain can be expected. The brain is the organ where SCs exert their psychotropic effects by binding to CB_1_ receptors (Giorgetti et al., 2020). Similar human brain levels were reported for MDMB-CHMICA (5.5 ng/g) (Gaunitz et al., 2018) and MAM-2201 (4.3 ng/g) (Saito et al., 2013), while low levels of 5F-MDMB-P7AICA in pigs were observed (0.1–0.33 ng/g) (Doerr et al., 2024). A comparatively high value of 19.6 ng/g (Hasegawa et al., 2015) was published for ADB-CHMINACA in a human fatal case. In a controlled pig study, the following brain concentrations were detected after single intravenous administration (200 μg/kg BW): 12 and 24 ng/g of Δ9-THC, 14 and 15 ng/g of RCS-4, and 20 and 32 ng/g of JWH-210 in cerebrum and cerebellum, respectively (Schaefer et al., 2017).

In cases 1 and 2, moderate ADB-BUTINACA concentrations were detected in **muscle** tissue (psoas, 2.0 and 1.5 ng/g), supporting earlier findings that muscle can serve as an alternative matrix in cases with putrefaction (Schaefer et al., 2017). Case 3 showed markedly higher levels (12 ng/g), possibly reflecting extensive tissue accumulation or chronic use. Elevated SC concentrations in deep compartments such as fat tissue may contribute to post-mortem redistribution into femoral blood, potentially biasing C/P ratios (Schaefer et al., 2020).

Tokarczyk et al. reported high **cerebrospinal fluid** (CSF) levels of 5F-MDMB-PICA and 4F-MDMB-BINACA, with cerebrospinal fluid/blood ratios exceeding 3.5 (Tokarczyk et al., 2022). In contrast, in the present study, cases 1 (1.0 ng/mL) and 3 (4.2 ng/mL) showed much lower cerebrospinal fluid/blood ratios of 0.25 and 0.5, respectively. This is consistent with the average drug ratio of 0.05–0.5 described by Engelhart et al. (Engelhart and Jenkins, 2007) and may also reflect shorter survival times after drug administration, during which lipophilic SCs preferentially accumulate in CNS tissue before CSF/blood equilibration. The detection of ADB-BUTINACA in CSF confirms that this compound can penetrate this hydrophilic matrix, supporting its utility as an alternative specimen.

In **vitreous humor**, ADB-BUTINACA concentrations were consistently low (0.33–2.1 ng/mL). As a closed and hydrophilic compartment, significant post-mortem redistribution or accumulation of lipophilic SCs is unlikely. Nevertheless, vitreous humor may serve as a useful matrix for comparing concentrations between cases due to its relative stability (Metushi et al., 2016; Hubbard et al., 2021; Walle et al., 2024). As expected, the LOD and LOQ of ADB-BUTINACA in vitreous humor were comparatively low compared to whole blood.

ADB-BUTINACA was detected in all matrices, with the highest concentrations in liver, lung, and kidney – organs involved in absorption, metabolism, and elimination. This distribution pattern is consistent with findings from animal studies in pigs and mice, where SCs also accumulated primarily in these organs (Schaefer et al., 2017; Wiebelhaus et al., 2012). This trend is consistent with the concentrations of the structurally similar ADB-CHMINACA, which was determined by standard addition in numerous post-mortem matrices. The highest concentration was detected in the liver (Hasegawa et al., 2015).

### 5.2 Toxicological significance score (TSS)

All three cases were mixed intoxications, requiring the consideration of concentrations of other detected drugs and medications when determining the cause of death (criteria: Supplementary Material, Supplementary Table S4). The classification of blood concentrations of ADB-BUTINACA as high, moderate, or low was based on the given concentrations from routine forensic casework from the Institute of Forensic Medicine Freiburg, derived from ante- and post-mortem blood and serum samples using external calibration. The median concentration of ADB-BUTINACA was 1.5 ng/mL. However, individual tolerance due to prior exposure may substantially influence the toxicological significance of these concentrations.

#### 5.2.1 Case 1

Ethanol and quetiapine were below levels regarded as toxic. Therefore, a contribution to the lethal outcome seems unlikely. The combination of the relatively high levels of ADB-BUTINACA (4.2 ng/mL) and pregabalin (25 μg/mL) in femoral blood have to be considered toxicologically significant and can explain death by mixed intoxication (Schulz et al., 2020). In this case, a TSS of 3 was assigned for ADB-BUTINACA due to the relatively low toxicity of pregabalin.

#### 5.2.2 Case 2

The morphine femoral blood concentration was in the therapeutic range, while the other listed substances (codeine, bupropion, olanzapine, promethazine) were at or below therapeutic levels, rendering a relevant contribution to death unlikely. Although ADB-BUTINACA (5.7 ng/mL, heart blood) and MDPHP (approx. 3.3 ng/mL femoral blood) were quantified, their toxicological significance is difficult to interpret due to lack of pharmacokinetic reference data. However, MDPHP should be well below the published toxicologically relevant range (Arillotta et al., 2024; Casati et al., 2025; Croce et al., 2025; Di Candia et al., 2022; Maida et al., 2025). Death was most likely the result of the combined effect of heroin and ADB-BUTINACA (Schulz et al., 2020). The presence of noscapine and papaverine in urine, in combination with a morphine-to-codeine ratio of approximately 3.8, indicates heroin use. The presence of the heroin metabolite 6-acetylmorphine as well as ATM4 and the respective glucuronide in urine (Chen et al., 2014) further confirmed heroin use. However, there have been reports of SCs being found as additives in heroin syringes (Ershad et al., 2020; King et al., 2022). This, together with the relatively high C/P ratio for morphine, can be used as justification for assigning a TSS of 1 to ADB-BUTINACA. Because of his history of consumption, the decedent may have been accustomed to the effects of ADB-BUTINACA with development of tolerance. This means that lower-moderate concentrations do not necessarily have toxic effects (Tai and Fantegrossi, 2014).

#### 5.2.3 Case 3

Although the blood ethanol level was high (approx. 2.9‰), chronic alcohol use may have induced tolerance, making alcohol alone an unlikely cause of death. The presence of the MDMB-4en-PINACA hydrolysis metabolite suggests prior ingestion, likely unrelated to death (Giorgetti et al., 2024b). THC and its metabolite THC-COOH were detected additionally. The concentrations determined for these analytes indicate consumption that occurred some time ago and, given the low toxicity of cannabis, cannot be considered a potential cause of death. The combination of high levels of ADB-BUTINACA (8.2 ng/mL) and alcohol is considered sufficient to explain death by mixed intoxication. Hence, a TSS of 2 was assigned to ADB-BUTINACA.

Co-exposure with other drugs is often seen in fatal cases involving SC. According to a review from Thomsen et al., co-consumption of SCs together with alcohol (39.4%) was highest in mortalities, followed by antipsychotics and antidepressants (34.3%) (Thomsen et al., 2024).

### 5.3 Stability assessment of ADB-BUTINACA in human whole blood

Besides post-mortem redistribution, drug stability is a critical factor in forensic interpretation. Stability depends on the chemical and metabolic properties of the substance, the matrix, and storage conditions, including duration of storage before analysis. In practice, deceased individuals are often discovered under conditions that hinder reliable analysis, for example, at room temperature or outdoors and in varying stages of decomposition. Bodies are typically stored at approximately 4 °C until autopsy, but under such circumstances, measured concentrations of SCs may not reflect the original peri-mortem levels. For accurate interpretation, factors such as circumstances of death, post-mortem interval, storage conditions of the body fluids and organs at −20 °C, and analyte instability must be considered (Abdelaal et al., 2024).

ADB-BUTINACA showed a clear temperature-dependent stability profile. At −20 °C, concentrations remained relatively stable over 84 days with minor fluctuations, highly likely due to changing matrix effects, indicating that deep-frozen storage can be regarded as well-suited for long-term preservation. At 4 °C, stronger variability and a gradual decline were observed, with an initial decrease to approximately 55% around day 14 followed by partial recovery to approximately 90% at day 84. This transient pattern likely reflects incomplete matrix equilibration of lipophilic ADB-BUTINACA in whole blood, partial degradation, adsorption to blood components during early storage, residual enzymatic activity, and matrix interactions that are not fully suppressed, indicating limited suitability of refrigerated storage. At 22 °C, concentrations continuously decreased, reflecting pronounced instability and rapid enzymatic and/or chemical degradation. This makes storage at room temperature unsuitable. The fluctuations can be attributed to matrix effects in whole blood, sample inhomogeneity, irreversible adsorption or binding to blood components, and analytical variability, while the overall temperature dependence may reflect both chemical lability (hydrolysis/oxidation) and enzymatic activity. The method’s LOD and LOQ are to be considered relatively high and are comparable to those published for ADB-BUTINACA in fly larvae as matrix (Blavier et al., 2026).

The present results show that samples containing SCs should be stored under deep-freeze conditions (−20 °C) to ensure stability of the substances. This corresponds with the recommendations of the current literature and the guidelines of the GTFCh (Djilali et al., 2022; Fort et al., 2017; Halter, 2020; Hess et al., 2017; Minakata et al., 2022; Paul et al., 2009).

Comparable stability studies confirm temperature-dependent SC degradation. Krotulski et al. investigated the stability of MMB-FUBINACA (FUB-AMB), 5F-MDMB-PINACA (5F-ADB), and 5F-MDMB-PICA in human whole blood over 35 days at room temperature, in a refrigerator, and in a freezer. Pronounced instability with formation of degradation products occurred at room temperature and refrigerator conditions, whereas freezer storage yielded only minor losses (Krotulski et al., 2021). Halter investigated the 30-day stability of 32 SCs in five human matrices: heart and femoral blood, serum (with potassium fluoride), and whole blood (with or without EDTA). Indazole derivatives containing a methyl ester in the bridging residue showed pronounced matrix- and structure-dependent instability, whereas amide analogs remained stable across all matrices (Halter, 2020).

Furthermore, degradation patterns of SCs in authentic post-mortem samples differ from those in spiked blood or serum. Halter reported faster hydrolysis in untreated cadaver material (e.g., heart or femoral blood) compared to stabilized whole blood, likely influenced by putrefaction processes (Halter, 2020). As the present study only examined untreated venous whole blood from living subjects, the results are not directly transferable to post-mortem blood samples, but indicate general trends for the storage of ADB-BUTINACA. The stability of SCs can be matrix-dependent.

In all three cases investigated herein, the bodies were found either immediately or within a few hours after death. The post-mortem interval, during which the bodies were stored at 4 °C, was 5 days in all cases. The samples analyzed were stored at −20 °C for two to 3 years. Therefore, interpretation of these findings must be approached with caution due to the limitations of this case series, including the small number of cases, the absence of pharmacokinetic, pharmacodynamic, and detailed toxicological data for ADB-BUTINACA, and the potential effects of the time lag between autopsy and analysis despite proper sample storage.

### 5.4 Metabolites

Although metabolism of xenobiotics is not the primary focus of post-mortem toxicology, metabolite identification remains relevant for analytical confirmation. Several phase I metabolites of ADB-BUTINACA have been described using *in vitro* and *in vivo* approaches. To complement our post-mortem investigations, we employed HepG2 cell incubations as an established *in vitro* model capable of generating both phase I and phase II metabolites (Zschiesche et al., 2024; Zschiesche et al., 2022), as well as a pHLM assay.

The high peak area ratios of the dihydrodiol metabolite correspond to descriptions of this compound in the literature as the main metabolite and primary biomarker (Kavanagh et al., 2022; Kronstrand et al., 2022; Zschiesche et al., 2025b). The monohydroxylated species (*N*-3-hydroxybutyl and indazole-OH) show high peak area ratios in urine and bile, supporting extensive renal and biliary elimination of these polar metabolites. ADB-INACA, a product of *N*-dealkylation, is merely a metabolic byproduct. However, it may also represent a byproduct of synthesis (Monti et al., 2025), which could not be confirmed due to the lack of samples of the used herbal mixtures or powders in the presented cases.

In case 2, in particular, our results generally showed high metabolite peak area ratios in urine and bile. This could indicate a longer survival period or chronic exposure to ADB-BUTINACA (Stephenson et al., 2024). Alternatively, enhanced CYP2C19 and CYP3A4 activity may have contributed to an increased metabolic turnover, consistent with an extensive metabolizer phenotype (Sia et al., 2021; Stephenson et al., 2024; Wu, 2011).

Metabolite peak area ratios were lowest in brain tissue. This can be explained by the greater permeability of the blood-brain barrier to lipophilic compounds and a ‘storage effect’ due to the relatively high fat content of brain tissue in combination with comparatively low metabolic activity in the central nervous system. However, it should be noted that matrix effects in this tissue are likely to be relatively large, and normalization to the parent substance cannot necessarily compensate for this. In accordance, LOD/LOQ of ADB-BUTINACA in the fat-rich matrix of fly larvae have shown to be relatively high (Blavier et al., 2026). Nevertheless, Schaefer et al. detected e.g., OH-RCS-4 in brain tissue of pigs (Schaefer et al., 2017).

The metabolite peak area ratios were also found to be comparatively low in vitreous humor. Substances diffuse from the blood into the interior of the eye primarily by passive diffusion through the blood-retinal barrier and the blood-aqueous humor barrier. For more polar metabolites, this diffusion is likely slower and less efficient because they must traverse these relatively lipophilic barrier structures, but once equilibrated they may preferentially reside in the aqueous vitreous compartment. Due to the absence of enzymes in the vitreous body, metabolic degradation is irrelevant in this compartment (Agrahari et al., 2016; Varela-Fernández et al., 2020).

The absence of glucuronides in liver and kidney tissue could be explained by the efficient excretion of these more polar metabolites. The formed conjugates are directly excreted into bile or urine instead of accumulating intracellularly. Additionally, post-mortem processes may favor rapid cleavage of glucuronides resulting in their aglycone components by post-mortem β-glucuronidase release. Methodologically, the high polarity of glucuronides likely makes efficient extraction from complex tissue matrices more difficult than from body fluids, resulting in increased matrix effects e.g., after work-up by protein precipitation (Ebuzoeme et al., 2021; Järvinen et al., 2022; Zamek-Gliszczynski et al., 2006).

The relatively low metabolite-to-parent peak area ratios in both *in vitro* systems are due to the very high chromatographic peak areas of ADB-BUTINACA (approx. 5 × 10^7^ counts per second) after incubation times of 30 min and 8 days, respectively, which can be regarded as a consequence of the low conversion rate. However, in pHLM incubation, monohydroxylation reactions dominate, despite the significantly shorter incubation time compared to HepG2 cells. This is probably due to the limited metabolic competence of HepG2 cells, particularly their low CYP activity and phase II enzyme expression compared to primary hepatocytes, or non-physiological cofactor excess (Gerets et al., 2012; Hart et al., 2010; Westerink and Schoonen, 2007). Additionally, HepG2 cells were incubated with a 50-fold lower concentration in comparison to the pHLM.

In general, however, these ratios are influenced by MS response factors, matrix effects, distribution properties, and post-mortem processes. Consequently, they reflect relative detectability and not absolute metabolic clearance rates.

As analytical approach for ADB-BUTINACA in post-mortem matrices, the detection of the parent compound can be considered sufficient. However, as with most SCs, the detection of metabolites, e.g., the compound specific biomarker dihydrodiol, is recommended for urine and bile fluid.

## 6 Conclusion

Data on the detailed toxicological assessment of fatal cases associated with synthetic cannabinoids are still scarce. In particular, little is known about the distribution patterns in the body or post-mortem redistribution processes. Despite the small number of cases, this series of fatalities involving ADB-BUTINACA provides for the first time a deeper insight into the distribution pattern of this potent and prevalent synthetic cannabinoid in typical post-mortem body fluids (femoral and heart blood, urine, gastric content, bile fluid, vitreous humor and cerebrospinal fluid) and organ tissues (brain, kidney, liver, lung and muscle). Macroscopically, only non-specific findings were present during autopsies of all three deaths (pulmonary and/or cerebral edema and blood-rich organs). As prescribed by many guidelines, the results of the present study point to the storage of ADB-BUTINACA-containing samples at −20 °C prior to analysis.

In addition to femoral and cardiac blood, lung, liver, muscle and kidney tissue can be recommended for the detection of ADB-BUTINACA in fatal cases. The SC ADB-BUTINACA likely contributed to the cause of death in all three mixed intoxication cases with a TSS of 1 (possible contribution to death) – 3 (primary cause of death). In general, the distribution pattern of ADB-BUTINACA follows a classic toxicokinetic model with concentration in the central nervous system, hepatic biotransformation to polar metabolites, and renal/biliary elimination. The metabolite profiles show that targeting ADB-BUTINACA metabolites seems not necessary, except in urine and bile fluid.

Further case studies dealing with fatal intoxications involving synthetic cannabinoids like MDMB-4en-PINACA and their distribution patterns are desirable and will provide further insights into this important topic.

## Data Availability

The original contributions presented in the study are included in the article/Supplementary Material, further inquiries can be directed to the corresponding author.
