# Supplementary material for: Synthetic cannabinoids in human post-mortem samples – ADB-BUTINACA and metabolites in three fatalities
Source: Front Toxicol. 2026 May 14;8:1826767. doi: 10.3389/ftox.2026.1826767 (PMC13215650; doi:10.3389/ftox.2026.1826767)
Supplement: Supplementary file 1 [file Table1.docx]

Synthetic Cannabinoids in Human Post-Mortem Samples – ADB-BUTINACA and Metabolites in Three Fatalities

Annette Zschiesche^1,2†^, Sophia Köpfler^1,3†^, Cora Wunder^4^, Annekathrin M. Keiler^5^, Johanna Görg^4^, Cleo P. Walz^4^, Barbara Fliss^4^, Tanja Germerott^4^, Volker Auwärter^1^*, Laura M. Huppertz^1^

^1^Institute of Forensic Medicine, Forensic Toxicology, Medical Center – University of Freiburg, Faculty of Medicine, University of Freiburg, Albertstr. 9, 79104 Freiburg, Germany

^2^Hermann Staudinger Graduate School, University of Freiburg, Hebelstr. 27, 79104 Freiburg, Germany

^3^Furtwangen University of Applied Sciences, Robert-Gerwig-Platz 1, 78120 Furtwangen, Germany

^4^Department of Forensic Toxicology, Institute of Legal Medicine, University Medical Center of the Johannes Gutenberg University, Am Pulverturm 3, 55131 Mainz, Germany

^5^Faculty of Biology, Environmental Monitoring and Endocrinology, TU Dresden University of Technology, Zellescher Weg 2b, 01217 Dresden, Germany

^†^These authors share first authorship

**Table S1**: Limit of quantification (LOQ) of LC-qToF-Screening and thresholds for quantification via GC-MS.

| **LC-qToF-MS** |  |
| --- | --- |
| **Analyte** | **LOQ** |
| Pregabaline | approx. 1000 ng/mL (quant 100 ng/mL) |
| Quetiapine | approx. 10 ng/mL |
| Opiates | approx. 25 ng/mL (quant 5 ng/mL) |
| Psychotropic drugs | approx. 10 ng/mL |
| Ketamine | approx. 10 ng/mL |
| **GC-MS** |  |
| **Analyte** | **Threshold** |
| Ethanol | from 0.1 per mille |
| THC | from 0.5 ng/mL |
| THC-COOH | from 2.5 ng/mL |

**Table S2**: Dilution factors for every post-mortem matrix and case, linear regression equation and linearity (R^2^: Regression correlation coefficient) were calculated using the mean values of the triplicates.

|  | **Dilution factor** | | | **Equation (linear regression)** | | | **R^2^** | | |
| --- | --- | --- | --- | --- | --- | --- | --- | --- | --- |
| **Matrix/Case** | **1** | **2** | **3** | **1** | **2** | **3** | **1** | **2** | **3** |
| **Femoral blood** | 5 | n.a. | 5 | y = 0.1906x+ 0.1634 | n.a. | y = 0.1815x + 0.3004 | 0.9964 | n.a. | 0.9973 |
| **Heart blood** | 5 | 5 | 5 | y = 0.1838x + 0.2891 | y = 0.1955x + 0.2241 | y = 0.1656x + 0.3658 | 0.9970 | 0.9974 | 0.9943 |
| **Urine** | n.a. | 1 | 1 | n.a. | y = 0.1869x + 0.0504 | y = 0.1734x + 0.0632 | n.a. | 0.9997 | 0.9975 |
| **Stomach content** | 1 | 1 | n.a. | y = 0.2030x + 0.6128 | y = 0.1936x + 0.3011 | n.a. | 0.9920 | 0.9978 | n.a. |
| **Bile fluid** | 5 | 5 | 5 | y = 0.2540x + 0.2560 | y = 0.1924x + 0.4939 | y = 0.2015x + 0.5346 | 0.9972 | 0.9985 | 0.9990 |
| **Brain tissue** | 6 | 5 | 6 | y = 0.1695x + 0.0884 | y = 0.2045x + 0.0224 | y = 0.236x + 0.1228 | 0.9979 | 0.9962 | 0.9993 |
| **Kidney tissue** | 5 | 6 | 6 | y = 0.1927x+ 0.1791 | y = 0.2145x + 0.0581 | y = 0.2236x + 0.2173 | 0.9984 | 0.9979 | 0.9990 |
| **Liver tissue** | 5 | 5 | 2 | y = 0.1634x + 0.4150 | y = 0.2200x + 0.1594 | y = 0.1801x + 2.4240 | 0.9932 | 0.9955 | 0.9859 |
| **Lung tissue** | 6 | 6 | 2 | y = 0.1803x + 0.0853 | y = 0.2268x + 0.1150 | y = 0.2024x + 0.1884 | 0.9944 | 0.9981 | 0.9989 |
| **Muscle tissue (psoas)** | 6 | 1 | 10 | y = 0.1860x + 0.0316 | y = 0.2836x + 0.2210 | y = 0.2180x + 0.2647 | 0.9994 | 0.9933 | 0.9985 |

n.a.: not available

Table S3: Optimized mass spectrometric parameters of the MRM ion transitions of ADB-BUTINACA and its metabolites. t_R_: retention time, Q1: *m/z* of the precursor ion, Q3: *m/z* of the fragment ion, DP: declustering potential, EP: entrance potential, CE: collision energy and CXP: cell exit potential.

| **Analyte** | **t_R_ [min]** | **Q1 [Da]** | **Q3 [Da]** | **DP [V]** | **EP [V]** | **CE [V]** | **CXP [V]** |
| --- | --- | --- | --- | --- | --- | --- | --- |
| ADB-BUTINACA | 4.41 | 331.21 | 201.1 | 56 | 10 | 35 | 8 |
|  |  |  | 286.19 | 56 | 10 | 21 | 12 |
|  |  |  | 145.02 | 56 | 10 | 57 | 16 |
| *N*-butyl 3OH | 2.27 | 347.20 | 217.1 | 50 | 10 | 34 | 13 |
|  |  |  | 302.1 | 50 | 10 | 22 | 16 |
|  |  |  | 145 | 50 | 10 | 55 | 13 |
| MonoOH indazole | 3.12 | 347.20 | 217.1 | 50 | 10 | 34 | 13 |
|  |  |  | 302.1 | 50 | 10 | 22 | 16 |
|  |  |  | 161 | 50 | 10 | 52 | 13 |
| *N*-Butanoic acid | 2.17 | 361.19 | 231.076 | 50 | 10 | 32 | 15 |
|  |  |  | 316.16 | 50 | 10 | 20 | 15 |
|  |  |  | 145 | 50 | 10 | 55 | 13 |
| ADB-INACA | 1.8 | 275.15 | 230.1 | 45 | 10 | 24 | 15 |
|  |  |  | 145 | 45 | 10 | 47 | 11 |
| Amide hydrolysis +Gluc | 3.65 | 508.20 | 286.2 | 62 | 10 | 35 | 15 |
|  |  |  | 201.1 | 62 | 10 | 42 | 13 |
|  |  |  | 332.2 | 62 | 10 | 27 | 17 |
| *N*-3OH butyl +Gluc | 1.47 | 523.20 | 217.101 | 63 | 10 | 48 | 13 |
|  |  |  | 302.2 | 63 | 10 | 39 | 15 |
|  |  |  | 145.01 | 63 | 10 | 67 | 10 |
| MonoOH Indazole +Gluc | 1.66 | 523.20 | 217.102 | 63 | 10 | 48 | 13 |
|  |  |  | 478.202 | 63 | 10 | 23 | 19 |
|  |  |  | 161 | 63 | 10 | 65 | 10 |
| Amide hydrolysis | 5.14 | 332.21 | 201.1 | 50 | 10 | 35 | 17 |
|  |  |  | 286.2 | 50 | 10 | 27 | 20 |
|  |  |  | 145 | 50 | 10 | 57 | 14 |
| Dihydrodiol (indazole) | 1.54 | 365.20 | 235.1 | 60 | 10 | 35 | 16 |
|  |  |  | 320.2 | 60 | 10 | 18 | 20 |
|  |  |  | 179 | 60 | 10 | 54 | 14 |
| Dihydrodiol (indazole) + Gluc | 0.89 | 541.30 | 496.2 | 65 | 10 | 25 | 19 |
|  |  |  | 235.1 | 65 | 10 | 52 | 11 |
|  |  |  | 411.1 | 65 | 10 | 29 | 16 |

Table S4: TSS criteria (Elliott et al., 2018) and case application.

| **TSS** | **Criteria** | **Case 1** | **Case 2** | **Case 3** |
| --- | --- | --- | --- | --- |
| **U** | Unclassified insufficient data to allow assessment | - | - | - |
| **1** | Possible contributory/low | - | **+** | - |
| **2** | Contributory cause/medium | - | - | **+** |
| **3** | Primary cause/high | **+** | - | - |

Elliott, S., Sedefov, R., Evans-Brown, M., 2018. Assessing the toxicological significance of new psychoactive substances in fatalities. Drug Test. Anal. 10, 120–126. https://doi.org/10.1002/dta.2225
